# Failure of volar locking plate fixation of an extraarticular distal radius fracture: A case report

**DOI:** 10.1186/1754-9493-4-19

**Published:** 2010-11-25

**Authors:** Jue Cao, Kagan Ozer

**Affiliations:** 1 Drexel University College of Medicine, Philadelphia, PA, USA; 2 Denver Health Medical Center, Associate Professor of Orthopedics, University of Colorado Denver, USA

## Abstract

**Background:**

Volar locking plates provide significant structural stability to the distal radius. Failure of a volar locked plating is a rarely reported complication in the literature.

**Case Presentation:**

A 40 year-old, obese female patient who presented with a displaced extraarticular distal radius fracture, underwent open reduction and internal fixation of the fracture using a volar locking plate. Radiographs taken at 10 weeks postoperatively showed failure of fixation with breakage of the four distal locking screws. A hardware removal was performed at 6 months, and the patient was then lost to follow-up. She presented again at 18 months after the first surgery, with significant pain, and radiographic signs of a radial collapse and a fracture-nonunion. A total wrist fusion was performed as the method of choice at that point in time.

**Conclusion:**

Volar locked plating represents the new "gold standard" of distal radius fracture fixation. However, despite the stability provided by locking plates, hardware failure may occur and lead to a cascade of complications which will ultimately require a wrist fusion, as outlined in this case report. Additional structural support by bone grafting may be needed in selected cases of volar locked plating, particularly in patients with a high risk of developing a fracture-nonunion.

## Background

Distal radius fractures are among the most common fractures of the musculoskeletal system. Functional outcome usually correlates well with maintenance of the radiographic reduction and the bony healing. In comminuted fractures of the distal radius, the use of bone grafts (autogenous, allograft, or in synthetic form) increases the structural stability at the fracture site and promotes bony union [1]. Following widespread use of volar locked plating systems, however, routine use of the bone grafts, particularly in the acute setting is believed to be unnecessary, even in comminuted fractures, since these fixation systems provide significant stability at the fracture site [2-4]. In fact volar locking plate failure due to nonunion of the distal radius is rare with limited number of reports in the English literature [5-7]. In this study, we report a case of nonunion of the distal radius leading to failure of the hardware on an obese patient.

## Case Report

**Figure 1:**
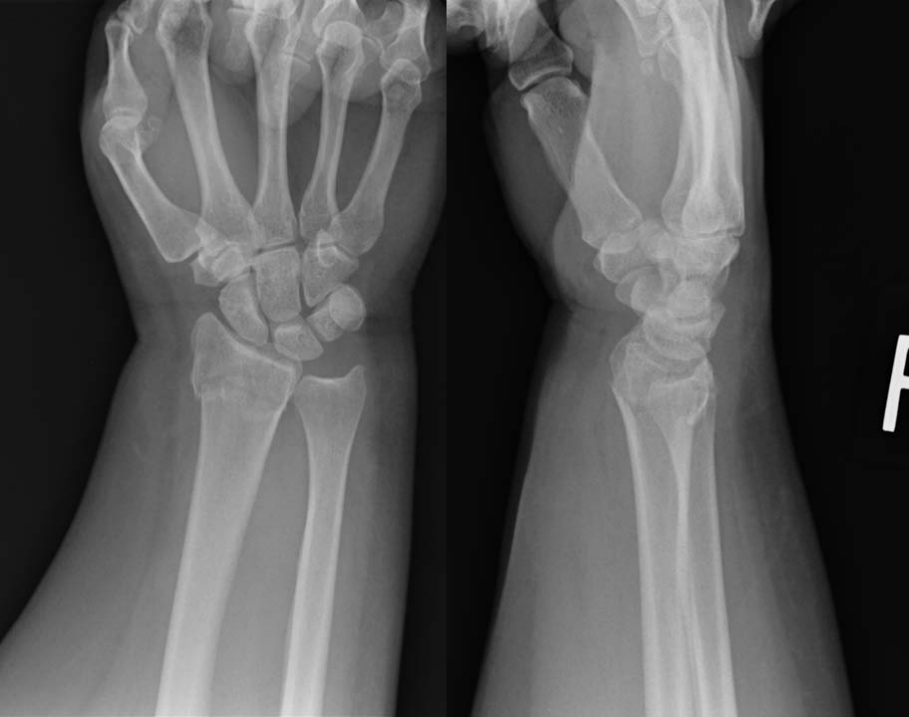
**Initial radiographic view of the right wrist following failure of reduction in 1 week**.

A 40-year-old, right hand dominant, unemployed, female presented to the emergency department with right wrist pain following a fall onto her right outstretched hand. She had a past medical history of smoking (30 pack/years), hypertension, adult obstructive sleep apnea, depression, bipolar disorder, and anxiety. Her body mass index (BMI) at that time was 39. Initial evaluations revealed a swollen wrist, no ecchymosis and a 2+ radial pulse. Radiographs demonstrated a right intra-articular distal radius fracture (AO, type 23-A3) in AP and lateral views. Following reduction, patient was placed in a sugar tong splint and was instructed to return for follow-up in one week. At 7 days, radiographs showed -20 degree of dorsal tilt (Figure 1). At that point, she chose to undergo surgery.

Patient underwent open reduction and internal fixation of the fracture using volar locked plating (Acumed, Aculock, Hillsboro, OR). The fracture was fixed with three 2.7 mm cortical screws on the shaft and four 2.3 mm locking screws distally. She was placed in a volar wrist splint for 10 days. Following suture removal, she received two sessions of formal physical therapy. At 4 weeks after the surgery, she had 30 degrees of wrist extension, 40 degrees of flexion, 10 degrees of radial deviation, 25 degrees of ulnar deviation, 60 degrees of supination, and 60 degrees of pronation. Her grip strength was 17 lbs on the right and 60 lbs on left.

**Figure 2:**
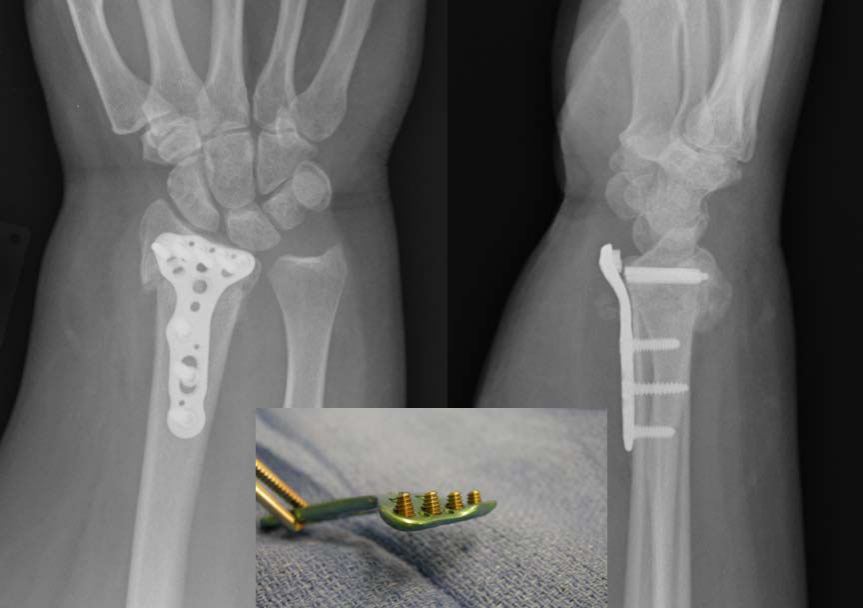
**Radiographs show collapse of the fracture following failure of screws**. Despite the collapse, the lateral radiograph showed neutral alignment of the distal radius articular surface with minimal shortening on the posteroanterior (PA) plane.

At 10 weeks after the surgery, she presented with new onset of pain following a popping sensation while trying to push a revolving door. She denied any falls or acute trauma, however admitted to have repeatedly used her arms to push herself up from a seated position. On physical examination, she had no obvious edema, erythema and ecchymosis. She did have tenderness to palpation over the distal radius and ulnar styloid process. She had decreased range of motion secondary to pain in flexion, extension, ulnar deviation, and radial deviation. The sensations to light touch in the distribution of radial, median, and ulnar nerves were intact. Radiographs showed 4 broken screws (Figure 2). No immediate action was taken to revise the surgery as radiographs showed only 2 mm shortening with neutral lateral tilt. Due to her tenderness, we placed her in a cast for 3 weeks, followed by a removable splint for 3 additional weeks.

**Figure 3:**
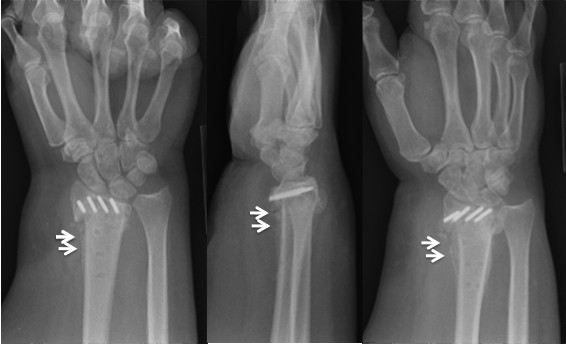
**Twelve months after the removal of the plate, patient continued to have symptoms**. Patient refused to have CT scan of her wrist as she had severe claustrophobia. Radiographic view obtained at the time showing bridging callus (arrows), however the diagnosis of nonunion was still not excluded before the surgery.

Six months following failure of the hardware, she continued to have ulnar sided wrist pain as well as impingement of the plate on the volar aspect of the wrist. She then elected to undergo plate removal. Following removal of the plate, she stayed relatively asymptomatic for 2 weeks and was lost to follow-up for 12 months. Eighteen months after the first surgery, she presented with significant pain, collapse of the radius relative to the ulna (4 mm), and a nonunion of the distal radius despite the bridging callus seen on the x-rays (Figure 3). She denied any major traumas since her fall, but her wrist pain had gradually worsened. Due to her significant pain, we took her to the OR for a revision surgery.

**Figure 4:**
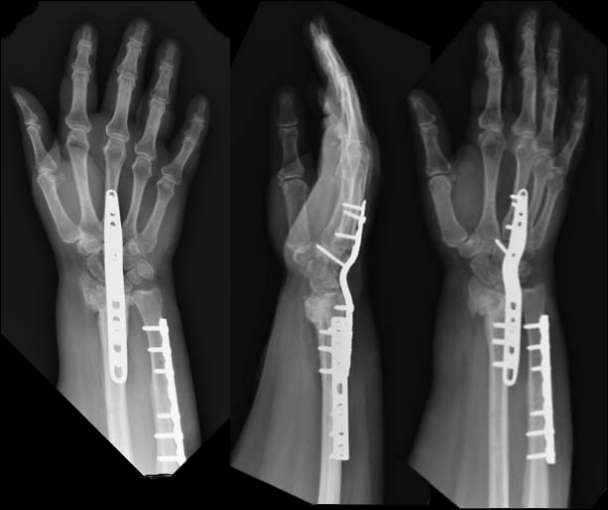
**Twelve months after the last surgery, radiographs show consolidation at the nonunion site**. Patient at this point is pain-free and has full forearm rotation.

During the surgery, a significant degree of bone loss and nonunion were noted at the fracture site. The distal end of the radius had a significant bone defect, approximately 2.5 cm in length, between the metaphyseal and the subchondral areas of the bone. In the absence of any bony support, the decision was made to span the join with an AO wrist fusion plate. This plate was preferred over conventional plates since it contained a combination of 3.5 mm screws on the radius and 2.7 mm screws on the metacarpal shaft. A total of 10 cc of demineralized bone matrix chips were placed at the fracture site. During the application of the fusion plate, we noted that the proximal carpal row was still impinging on the head of the ulna. Therefore, we decided to perform an ulnar shortening osteotomy at the same time. Final radiographic views obtained 12 months after the wrist fusion surgery is shown on Figure 4.

## Discussion

Repetitive bending forces accumulated within a distal radius plate over time may lead to hardware failure if bone healing is delayed. As in our case, the initial delayed healing along with repetitive use of the wrist likely caused an increase in force accumulation in the implant over time and once the force accumulated surpassed the screw strength, failure of the hardware was inevitable. Although nonunions of the distal radius with plate failure are relatively rare, our case is a good example of the race between bone healing and hardware failure [4].

Patient's co-morbidities and the repetitive minor trauma to her wrist might have contributed to the initial failed healing as well as the hardware failure. History of smoking, diabetes, obesity, and having an open fracture with soft tissue injury are recognized risk factors for distal radius nonunion [5-7]. Our patient had a long history of smoking, which has been shown to lengthen the healing time as well as promoting non-unions in tibial fractures [8]. In a monozygotic twin discordant tobacco use study, Hopper and Seeman discovered a 5% to 10% bone density deficit in patients who smoked compared with those patients who were nonsmokers [9]. Also, in a report of five cases of non-unions of distal radius fractures, it was found that all five patients were heavy smokers [6]. Smoking can have an adverse effect on bone density and delaying bone healing, which might explain the delayed union time for our patient [10]. Another factor leading to delayed union may be obesity [11]. Our patient's plate failure and previously reported locked plate failures have both occurred on morbidly obese individuals [5]. A report of 12 fracture cases, it was documented that there is a correlation between morbid obesity and non-union of the distal radius [7]. Another factor contributing to the failure is the repetitive use of the wrist following surgery. Our patient admitted to having used her wrist multiple times to push herself out of the chair. By pushing herself up from a seated position every time, the obese patient likely produced immense amount of force on her distal radius fragment and distal plate screws. Overtime, the plate-screw interface failed given the lack of support from the bone [7].

As for the placement of the plate on the bone, it has been shown that there is a significant decrease in axial stiffness and tensional rigidity that becomes evident at a distance of 5 mm between plate and bone [12]. Also poor contact between the plate and the anterior cortex of the distal radius is a factor leading to plate failure [5]. In our case, post-operative X-rays showed a 3 mm gap between the most distal section of the volar plate and the distal radius. In this situation, the screws may have had to take on more torque and stress over time.

Another factor that may have strengthened the fixation is the number and the diameter of screws used to fix the fracture. We only used 4 distal locking screws. The plate however was designed to accommodate up to 7 screws distally. Increasing the number of distal fixation screws could potentially have improved the stability and provided more time for the fracture to heal. On the other hand, the surgeon was also unable to control the screw diameter. Commercially available distal radius plating systems offer a range of distal screw diameters between 2.3 and 2.7 mm. Although, there have not been any biomechanical studies comparing the relationship between the number of distal screws/screw diameters and force to failure ratio, all 4 screws in our case failed at the shaft-bone juncture (Figure 2). This is known as the weakest point in force transfer between the volar plate and the distal radius fragment [3,13,14]. Therefore, increasing the number of screws and/or their diameters could potentially have increased the stability of the construct.

Following significant collapse of the distal radius, we chose to perform a total wrist fusion over alternative methods such as a radioscapholunate fusion. This decision was mostly based on the fact that this patient had poor bone quality at the time of surgery and the need to have a stable construct which will withstand a great deal of deforming forces such as when she pushes herself out of a chair. In the absence of good bony support, the three screws that potentially would have been placed on scaphoid and lunate in a radioscapholunate fusion would not have been able to provide the kind of stability this patient required. We therefore chose to lengthen the lever arm of the fixation by passing the wrist joint and performing a total wrist fusion.

## Conclusion

Although, delayed union and nonunions of distal radius fractures are rare, failure of the hardware is possible even with the use of volar locking plates. Primary bone grafting especially in patients with co-morbidities such as a history of smoking, and morbid obesity, may be advisable.

## Consent

Written informed consent was obtained from the patient for publication of this case report and any accompanying images. A copy of the written consent is available for review by the Editor-in-Chief of this journal.

## Competing interests

The authors declare that they have no competing interests.

## Authors' contributions

JC was involved in acquisition of the data and drafting of the manuscript. KO made substantial contributions to the conception, design, drafting and final approval of the manuscript. All authors read and approved the final manuscript.
